# Experimental Study on Cementitious Composites Embedded with Organic Microcapsules

**DOI:** 10.3390/ma6094064

**Published:** 2013-09-16

**Authors:** Xianfeng Wang, Feng Xing, Ming Zhang, Ningxu Han, Zhiwei Qian

**Affiliations:** 1 Guangdong Provincial Key Laboratory of Durability for Marine Civil Engineering, College of Civil Engineering, Shenzhen University, Shenzhen, Guangdong 518060, China; E-Mails: zhangmingjob@sina.com (M.Z.); nxhan@szu.edu.cn (N.H.); 2 Faculty of Civil Engineering and GeoSciences, Delft University of Technology, Delft 2600 AA, The Netherlands; E-Mail: z.qian@tudelft.nl

**Keywords:** self-healing concrete, organic microcapsules, permeability, recovery rate, healing effect

## Abstract

The recovery behavior for strength and impermeability of cementitious composites embedded with organic microcapsules was investigated in this study. Mortar specimens were formed by mixing the organic microcapsules and a catalyst with cement and sand. The mechanical behaviors of flexural and compression strength were tested. The results showed that strength could increase by up to nine percent with the addition of a small amount of microcapsules and then decrease with an increasing amount of microcapsules. An orthogonal test for investigating the strength recovery rate was designed and implemented for bending and compression using the factors of water/cement ratio, amount of microcapsules, and preloading rate. It is shown that the amount of microcapsules plays a key role in the strength recovery rate. Chloride ion permeability tests were also carried out to investigate the recovery rate and healing effect. The initial damage was obtained by subjecting the specimens to compression. Both the recovery rate and the healing effect were nearly proportional to the amount of microcapsules. The obtained cementitious composites can be seen as self-healing owing to their recovery behavior for both strength and permeability.

## 1. Introduction

Concrete is widely used worldwide in construction because of its low cost, high compressive strength, good compatibility with steel, and the ease of which it can be designed. However, crack formation is prone to present in concrete structures owing to its low tensile strength, which may lead to a durability problem and may also affect its serviceability. To prevent such deterioration, inspection of cracks and subsequent repair are usually carried out for concrete structures, thus various techniques and methods have been developed. However, continuous inspection and maintenance can be difficult to implement when cracks are not visible or accessible—for example, for underground structures or in the case of infrastructure in continuous service such as highways. Moreover, a considerable amount of labor is required and costs related to repair work may amount to half of the annual construction budget [1]. In contrast, in nature, damage can usually be self-healed. Thus, structures that mimic nature and self-healing could be of great interest.

Van Breugel [2] summarized the research history of self-healing phenomena in cementitious materials. Wu* et al.* [3], Joseph* et al.* [4], and Mihashi and Nishiwaki [5] presented reviews on self-healing in cementitious materials and engineered cementitious composite as a self-healing material. Self-healing is actually a well-known phenomenon for concrete because it possesses some natural healing properties. As a result of further hydration of unhydrated cementitious components or formation of calcium carbonate by carbonation of calcium hydroxide, cracks may heal after some time. However, autogenous healing is only effective when water is available and it is dependent on the amount of rest un-hydrated binding materials. Therefore, concrete must be modified in an artificial way to develop self-healing properties.

Though there have been classification schemes presented in the review papers, from the point of view of the authors, at present, there are two ways to achieve a self-healing function for concrete structures: One is implemented at the material level and the other is implemented at the structural level.

At the material level, one can make use of bacteria, microcapsules, or expansive agents and mineral admixtures. Usually at this level, self-healing occurs passively. That is, one mixes the healing agent uniformly into the concrete matrix. When a crack propagates to meet the healing agent, it will start to function and fill the crack face.

Using the microbiological method, Gollapudi* et al.* [6] presented a pioneering work in which a kind of ureolytic bacteria was incorporated to aid the precipitation of calcium carbonate (CaCO_3_) in the microcrack region. Tittelboom* et al.* [7] used bacteria manually healing cracks, which were created in two ways: one was standardized, and the other had more realistic cracks. Tittelboom* et al.* [7] carried out the tests by the use of *Bacillus sphaericus* bacteria of six distinguished strains, which were able to precipitate CaCO_3_ by conversion of urea into ammonium and carbonate. The bacterial degradation of urea locally increased the pH and promoted the microbial deposition of carbonate, as calcium carbonates in a calcium rich environment. They made a comparison between the microbial repair technique and traditional methods used to repair cracks in concrete by means of water permeability tests, ultrasound transmission measurements and visual examination. It was realized that pure bacteria cultures were not able to bridge the cracks. However, when bacteria were protected in silica gel, cracks were filled completely. Jonkers* et al.* [8,9,10,11] investigated the potential of bacteria to repair cracks occurring in concrete. In their study, a specific group of alkali-resistant spore-forming bacteria related to the genus Bacillus was selected. Bacterial spores directly added to the cement paste mixture remained viable for a period up to four months. They suggested that bacterial cement stone specimens appeared to produce substantially more crack-sealing minerals than control specimens; thus the potential application of bacterial spores as a self-healing agent appeared promising. However, there were still some key processes that should be determined or clarified in advance; these included compatibility with concrete, function trigger timing, and control of reaction level.

Another method at the material level involves the use of admixtures. Kishi* et al.* [12,13] developed a self-healing concrete using a combination of an expansive agent, geomaterials and chemical admixtures for practical industrial application. Their results showed that a concrete crack was significantly self-healed after 28 days of re-curing. A crack of width 0.15 mm was self-healed after re-curing for three days and the crack width decreased from 0.22 to 0.16 mm after re-curing for 7 days, and it was almost completely self-healed by 33 days. Kishi* et al.* [12,13] suggested that the phenomenon mainly resulted from the effects of swelling, expansion, and recrystallization. This approach required a supply of water or at least moisture, but they indicated that the utilization of appropriate dosages of geomaterials has a high potential for repairing cracked concrete. Taniguchi* et al.* [14] developed a self-healing concrete that incorporates fly ash for maintaining the durability of concrete structures in cold regions. In their study, sand was replaced with fly ash comprising 15% of cement by weight. After undergoing accelerated freeze-thaw cycles until their relative dynamic modulus of elasticity was reduced to 80%, specimens were cured in water at 40 °C for 28 days as a second curing. The reduction of the carbonation entrainment rate was found to be significant in the case of concrete containing fly ash and entrained air. The problem with this method was controlling the expansive agent. Unexpected expansion could occur often in cementitious matrices if the restraints were not appropriately set; this could lead to immature failure of the matrices, resulting in lower strength.

Encapsulating microcapsules into the matrix was also used for self-healing of materials. First, the microcapsules incorporating the healing agent were mixed uniformly into the concrete matrix; when a crack initiated and propagated, the embedded microcapsules ruptured under stresses and the healing agent was released into the crack plane through capillary action to achieve the healing function. This passive method has attracted much attention since the paper in *Nature* by White* et al.* [15] in 2001 in which the method was applied to polymer materials. Xing* et al.* [16] developed a self-healing technique using organic microcapsules for cement paste. In their study, the integrity of organic microcapsules was maintained during the making of the cement paste, and the microcapsules ruptured when cracks passed through them. An element analysis provided definite proof of the healing phenomenon on the crack faces. The effect of various proportions of microcapsules was also investigated. Results suggested that, although the method needed to be further developed in the future, encapsulation looked to be a promising approach to self-healing. Yang* et al.* [17] developed a self-healing system of microcapsules with an oil core and silica gel shell. Methylmethacrylate monomer and triethylborane were selected as the healing agent and the catalyst, respectively. The microcapsules were dispersed in fresh cement mortar along with carbon microfibers. The self-healing effect was evaluated by using permeability measurements along with a fatigue test under uniaxial compression cyclic loading and was further confirmed by surface analytical tools and a field emission scanning electron microscope.

The other way to achieve self-healing is implemented at the structural level. This can include the use of hollow fibers or shape memory material fibers. In this method, structural or location design is needed, sensor devices may also be arranged, and the self-healing can be active and/or passive. One of the earliest studies in the field of concrete engineering was carried out by Dry [18,19]. In her study, an adhesive agent contained in hollow brittle glass fibers served as the repairing chemical. Later, Dry [20] designed internally placed encapsulators containing sealants, adhesives, and waterproofing to release the chemicals where and when they were needed. Li* et al.* [21] used cyanoacrylate enclosed in capillary tubes sealed with silicon. Similar work was conducted by Mihashi* et al.* [22] and Joseph* et al.* [23]. Hollow tubes were placed inside a cementitious matrix, with one end linked to the supply of healing agent and the other end sealed. Nishiwaki* et al.* [24] proposed a self-healing system consisting of a self-diagnosis composite, healing agent, and a heat-plasticity organic film pipe. The self-diagnosing composite could detect the initiation of cracks and was functionalized as a heating device that could increase the electric resistance around cracks and heat the damaged parts selectively through electrification. Then, a heat-plasticity organic film pipe containing a one-component epoxy resin embedded in the concrete next to the heating device was melted, releasing the healing agent to fill in the cracks.

Sakai* et al.* [25] proposed a crack-closure system using a shape memory alloy (SMA). They used a SMA as the main reinforcing bars for concrete beams to enable large cracks under loading to be mechanically closed after unloading. Jefferson* et al.* [26] developed a similar crack-closure system for cementitious materials using shrinkable polymer tendons. The system involved the incorporation of unbonded preoriented polymer tendons in cementitious beams. Cracks were closed through thermal activation of the shrinkage mechanism of the restrained polymer tendons.

In this study, a self-healing system of mortar was developed using organic microcapsules incorporating a healing agent. To achieve self-healing function, healing agent embedded in the matrix needs to be intact until trigger excitation. Encapsulation of healing agent with microcapsules is a passive and appropriate way. As mentioned above [16], organic microcapsules were developed in Guangdong Provincial Key Laboratory of Durability for Marine Civil Engineering. Taking advantage of this, organic microcapsules, together with a catalyst, were mixed uniformly into the mortar to make a self-healing material. Damaged specimens were obtained by using compression at different loading levels. The flexural and compressive strengths were investigated for specimens with organic microcapsules and reference specimens. The chloride ion permeability was tested. The healing effect was investigated by using mechanical and permeability indices.

## 2. Experiments and Discussion

### 2.1. Microcapsules

Organic microcapsules were synthesized at the Guangdong Provincial Key Laboratory of Durability for Marine Civil Engineering, Shenzhen University. The shell material is urea formoldehyde (UF), and the core healing agent is epoxy.

**Figure 1:**
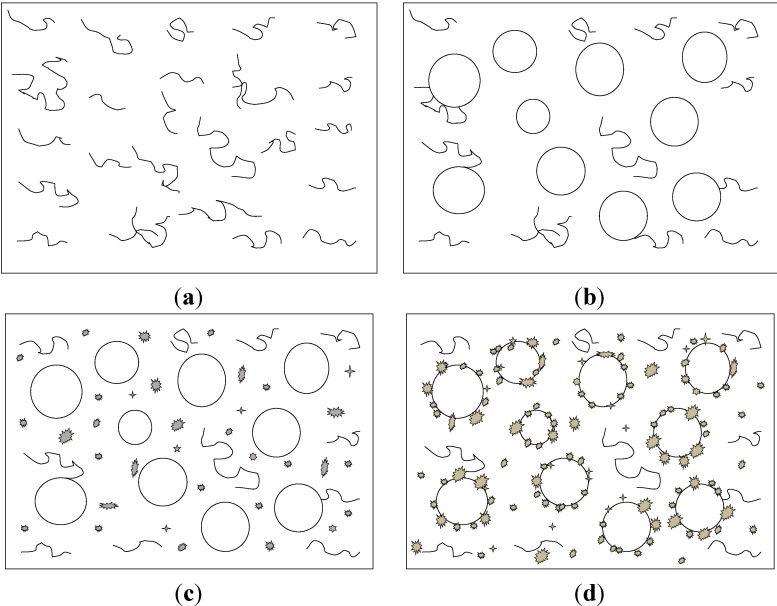
Synthesis stages of organic microcapsules. (**a**) Pre-polymer prepared; (**b**) Core material dispersed into droplet; (**c**) Pre-polymer polycondensated and (**d**) Polycondensated pre-polymer deposited on core.

The materials used for synthesis were urea (Analytical Reagent: AR), formaldehyde solution (AR), triethanolamine (AR), butyl glycidyl ether (BGE), epoxy resin E-51, and sulfuric acid. There were basically four stages in forming organic microcapsules as shown in Figure 1: (a) Urea was dissolved in 37 wt % formoldehyde aqueous solution at 1:1.5 molar ratio; the mixture was reacted under stirring; (b) Core material was dispersed into droplet; BGE was added to epoxy adhesive E-51; the epoxy was then added to the obtained UF pre-polymer; (c) Pre-polymer was polycondensated and (d) Sulfuric acid was added slowly to adjust the pH to a value of 2–3, and then the reaction was maintained for over 2 h. Polycondensated pre-polymer was deposited on the core to form microcapsules. Finally, the microcapsules were washed with water and dried 24 h at 60 °C in drying cabinet, making them ready for use. The details of the synthesis can be found in reference [16].

**Figure 2:**
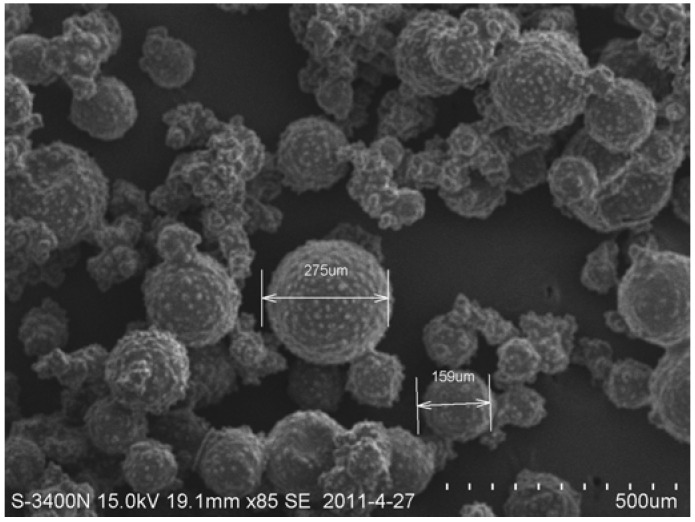
Organic microcapsules under a scanning electron microscope (SEM).

The diameter, shell thickness, and surface texture of the organic microcapsules could all be adjusted by controlling the stirring speed in the stages. The organic microcapsules under a scanning electron microscope [SEM; S-3400N(II), Hitachi High-Technologies Corp., Tokyo, Japan] are shown in Figure 2. It was seen that the shape of the organic microcapsules was nearly regularly spherical. Nearby, small amounts of remaining epoxy were also seen, which were irregular and dispersed around microcapsules. The surfaces of the organic microcapsules were seen to be complete and dense. One could also see that there were microspherical processes occurring on the surfaces of the organic microcapsules, roughening the surfaces and facilitating the formation of a good interface with the cement hydration products.

**Figure 3:**
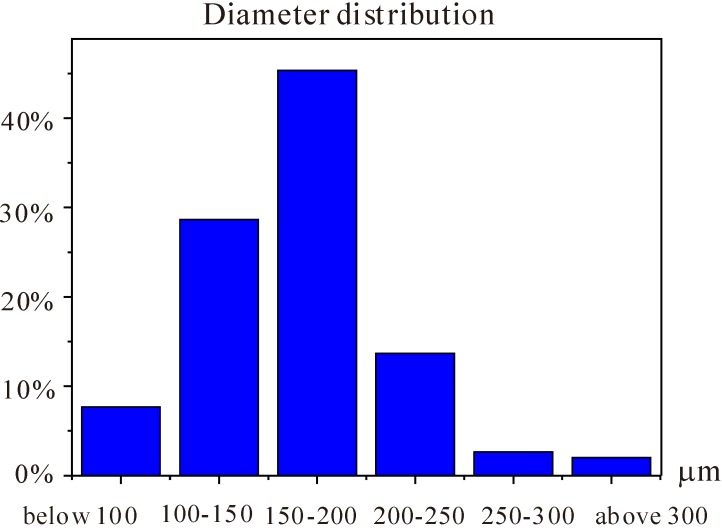
Diameter distribution of the organic microcapsules (in micrometers).

To observe the obtained microcapsules, some pictures were taken by optical microscope and scanning electron microscope (SEM), from which a randomly selected sample of 300 microcapsules was used to determine the diameter distribution as shown in Figure 3. The average diameter was 166 μm, and the standard deviation was 47 μm. The maximum and minimum values were 309 and 73 μm, respectively.

**Figure 4:**
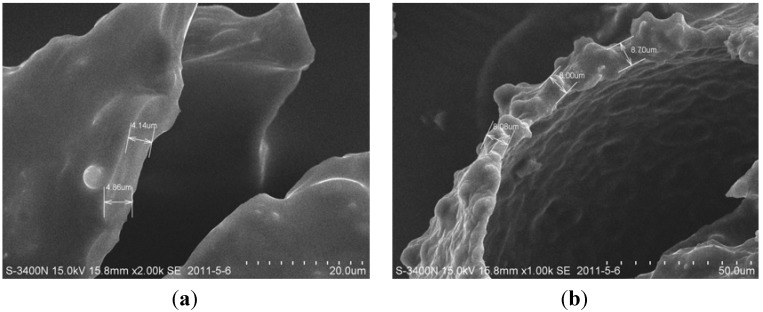
Shell thickness of organic microcapsules.

The shell thickness of the organic microcapsules was tested using SEM pictures when microcapsules were ruptured. The ruptured microcapsules could be obtained by grinding or later be found at the fracture interface of specimens. It was found that the shell thickness was about 4–8 μm, as shown in Figure 4.

### 2.2. Mortar Specimens

Two kinds of mortar specimens were prepared for the tests of mechanical and chloride ion permeability behaviors, respectively. The materials used were (1) cement (China Portland cement GB-175-2007 PII 42.5R [27]), (2) sand (China Xiamen GB/T17671-1999 ISO [28] standard sand), and (3) an encapsulated catalyst [2-heptadecylimidazole (MC120D)] together with the organic microcapsules for the composite design. According to the chemistry, 2-heptadecylimidazole could react with the epoxy (core healing agent) at room temperature [29].

The mortar specimens were prepared by using the following procedure: First, the cement, microcapsules, and MC120D catalyst were mixed together; then water and the admixture were placed in the mixer; next, the mixer was started and sand was added uniformly; the mixture was slowly stirred for 3 min, after which the mixer was paused for 30 s, then stirring was resumed for 3 min. Prismatic specimens with dimensions of 40 mm × 40 mm × 160 mm were cast for mechanical behavior tests. The cylinder specimens with dimensions of ϕ 100 mm × 100 mm were cast and later cut into ϕ 100 mm × 50 mm pieces before chloride ion permeability tests. The specimens were demolded after 24 h and cured for 28 days under the same condition as the standard mortar tests (temperature 20 °C, humidity >90%).

### 2.3. Mechanical Behavior

#### 2.3.1. Variation of Mechanical Behavior Resulting from the Addition of Organic Microcapsules

First, the effects of the organic microcapsules on the original strength of the mortar specimens were investigated. The mix proportions of the composite specimens are shown in Table 1, in which the water/cement ratio (W/C = 0.45, 0.50 and 0.55) and the microcapsule content (0%, 3%, 6% and 9% to cement mass) were the considered factors. In this research, 150 g of cement and 450 g of sand were used in each specimen. The amount of catalyst MC120D used was half the amount of organic microcapsules.

**Table 1:** Specimens for mechanical tests.

Sample No.	1	2	3	4	5	6	7	8	9	10	11	12
Sample Name	0.45–0	0.45–3	0.45–6	0.45–9	0.5–0	0.5–3	0.5–6	0.5–9	0.55–0	0.55–3	0.55–6	0.55–9
W/C	0.45	0.45	0.45	0.45	0.5	0.5	0.5	0.5	0.55	0.55	0.55	0.55
Microcapsule content (to cement mass)	0%	3%	6%	9%	0%	3%	6%	9%	0%	3%	6%	9%

The testing machine was type RGM-4010 (REGEL Corp., Shenzhen, China). The testing method was based on a standard [28]. Three specimens were grouped as one sample in the flexural strength test, which was conducted using the three-point bending method. The distance between two supports was 100 mm and the loading speed was 1 mm/s. After the flexural strength test, the mortar specimens were broken into two parts at the middle surface. Then, the six half-specimens were used for the compressive strength test. The area under compression for each specimen is 40 mm × 40 mm. The loading speed for the compression test was 2.4 kN/s. Average values for respective flexural and compressive strength tests were used for each sample.

For a three-point bending test, the flexural strength *R_F_* was given by [28]:

(1) $$R_{F}=\frac{3FL}{2bd^{2}}$$

where *F* denotes the load at the fracture point, *L* represents the length of the support span (100 mm in this case), and *b* and *d* signify the width and height of the specimen intersection, respectively. The compression strength was the maximum compression load divided by the loading area (40 mm × 40 mm).

**Figure 5:**
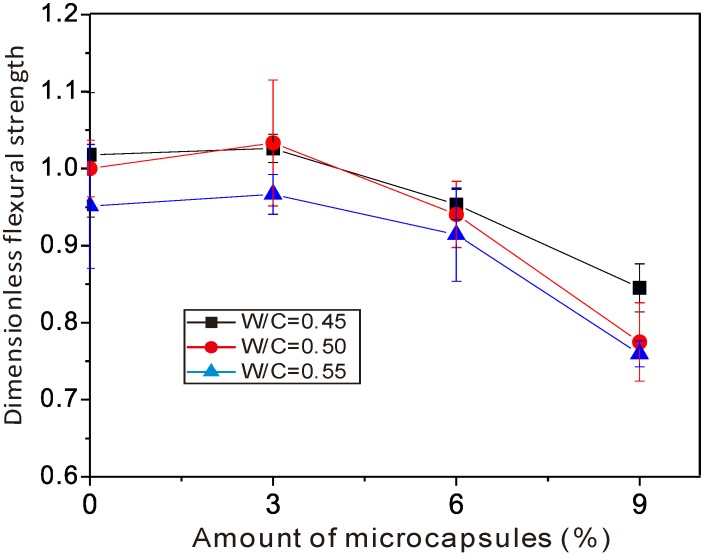
Variation of the flexural strength* versus* the amount of organic microcapsules.

**Figure 6:**
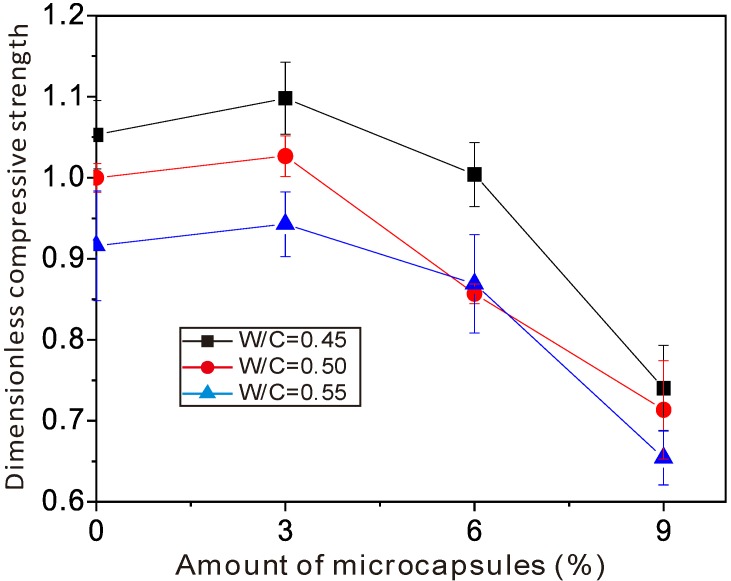
Variation of the compressive strength* versus* the amount of organic microcapsules.

Figure 5 and Figure 6 show the variation of flexural strength and compressive strength, respectively,* versus* the amount of organic microcapsules. The horizontal axes are the amount of microcapsules in percent (%). The vertical axes are the flexural and compressive strengths, respectively, divided by the ones for sample No. 5, that is, W/C = 0.5, and without microcapsules. Herein, for convenience of expression, sample No. 5 was treated as a reference. Its flexural and compressive strengths were 8.40 and 48.9 MPa, respectively. The error bars in the figures indicated the standard deviation for each data, in which the maximums in Figure 5 and Figure 6 were 0.08 and 0.06, respectively.

As was seen from the figures, there was a tendency for the organic microcapsules to induce a decrease of both flexural and compressive strengths overall, when the amount of microcapsules was 9%, the flexural and the compressive strengths decreased by 25% and 35%, respectively. With a small amount of organic microcapsules (about 3%), neither flexural nor compressive strength decreased—or they could even increase up to 3% for flexural strength and 9% for compressive strength. It is known that [30] Young’s modulus of the microcapsule wall (UF) locates under 10 GPa, whereas for the cement mortar, it ranges from 10 to 30 GPa. Lower Young’s modulus and weaker interface strength should make organic microcapsules behave as defects in the matrix. That is true for large amounts of microcapsules, while it should be also noted that in cement mortars, the size of sand is larger than that of microcapsules. Small holes could be generated while making the specimens. From the diameter distribution of organic microcapsules, it is supposed that the increase of strength for small amounts of organic microcapsules should come from the microfiller effect in the mortar specimens. However, with further increases in the microcapsule amount, more defects may be formed and the strength decreases. One also sees wide gaps between curves in Figure 6 compared to that in Figure 5, which means that the water/cement ratio affected the compressive strength more than the flexural strength.

#### 2.3.2. Strength Recovery Rate Resulting from Self-Healing

To investigate the self-healing effect on the strength recovery rate, the experiments were designed to investigate three factors: W/C, amount of organic microcapsules, and preloading rate, in which the preloading applied to the prismatic specimens was used to obtain initial damage and induced cracks for investigating the self-healing phenomenon. The binder-sand ratio was 1:3 as well. The reference groups were tested to obtain the original strengths (column 5 in Table 2), from which preloading rates were determined. Thus, each reference group corresponded to one combination of parameters. In the experiments, three levels were considered for each factor (*i.e.*, W/C = 0.45, 0.50 and 0.55; amount of microcapsules = 0%, 3% and 6%; and preloading rate = 30%, 50% and 70%). To reduce the number of tests and to make the analysis easier, an orthogonal test plan was set up and carried out to investigate the strength recovery rate as a result of self-healing. The strength recovery rate was defined by:

(2) $$\text{recovery rate}=\frac{\text{strength after healing}}{\text{original strength}}\times 100\%$$

where the original strength denotes the specimen strength at 28 days, and the strength after healing was obtained by using the following steps: (1) Apply the prescribed preloading to the specimen at 28 days; (2) Leave the specimen curing for 3 days (curing condition: temperature 20 °C, humidity >90%); (3) Test the strength.

As mentioned above, because there were three factors (W/C, amount of microcapsules, preloading rate) and each factor had three levels, an *L*_9_(3^4^) array, as shown in Table 2, was used [31]. Herein, the three factors were considered independent of each other. Column 4 was used for error analysis. Only a total of nine tests were needed. Column 5 was filled with the specimen original strengths, which were the results of the reference groups. Column 6 was used for the specimen strength after 3 days healing. The strength recovery rates occupying Column 7 were computed from Equation (2). *K*1, *K*2, and *K*3 were the average values of the recovery rates for the corresponding levels and factors,* i.e.*, for *K_ij_*, the first index *i* represents the level, while the second index *j* stands for the factor A (W/C), B (Amount of microcapsules), C (Preloading rate) and the error (D). *R* represents the range of each factor (maximum value—minimum value).

To investigate the reliability of the test results and the role of each factor, an analysis of the variance was carried out in Table 3. The column “Resource” lists the factors; the column “Deviation summation* SS_j_*” was computed by [31]:

(3) $$SS_{j}=\frac{1}{r}\sum_{i=1}^{m}K_{ij}^{2}-\frac{T^{2}}{n}$$

where *r* = *n*/*m*, with *n* and *m* representing the total number of tests and number of levels of each factor, respectively, *K_ij_* are the average values in Table 2, and *T* is the summation of the nine recovery rates in the tests. The freedom of each factor was obtained by the level value minus one. The average deviation summation *MS_j_* was determined by the deviation summation *SS_j_* divided by freedom *f.* The *F* value was defined by the average deviation *MS_j_* of each factor divided by that of the error, which represents the influence of each factor on the recovery rate. The critical *F* values, *F*α (P (F > *F*α) = α) can be found in [31]. By comparing *F* value to *F*α, it can be determined how remarkable its influence is. The probability was (1 − α) × 100%. Besides, the column contribution was obtained from [31]:

(4) $$\rho_{j}=\frac{SS_{j}-f_{j}MS_{e}}{S_{T}}\%$$

where *S_T_* is the total deviation summation. By comparing values of *F* to those of *F*α and knowing the contribution ρ, contribution of each factor was determined: The biggest one was the amount of microcapsules (81.16%), then the preloading (12.62%); the smallest one was W/C (3.64%), which meant that the amount of microcapsules could mostly affect the recovery rate, with the influence of preloading and W/C being relatively weak. The error of the contribution was 2.58% as seen in Table 3, indicating that the results should be acceptable.

**Table 2:** Orthogonal experiments for flexural strength test: factors, levels, and results.

No.	A (W/C)	B (Amount of microcapsules)	C (Preloading rate)	D (Error)	Original strength (MPa)	Strength after healing (MPa)	Recovery rate
1	0.45	0%	30%	1	9.1	8.9	97%
2	0.45	3%	50%	2	8.9	9.5	107%
3	0.45	6%	70%	3	7.9	8.9	113%
4	0.5	0%	50%	3	8.3	8.4	101%
5	0.5	3%	70%	1	8.2	8.6	104%
6	0.5	6%	30%	2	7.6	9.3	122%
7	0.55	0%	70%	2	7.8	6.7	86%
8	0.55	3%	30%	3	7.7	8.2	106%
9	0.55	6%	50%	1	7.1	8.5	119%
*K*1	106%	95%	109%	107%	-	-	-
*K*2	109%	106%	109%	105%	-	-	-
*K*3	104%	118%	101%	107%	-	-	-
*R*	5%	23%	8%	2%	-	-	-

**Table 3:** Analysis of variance for flexural strength tests.

Resource	Deviation summation *SS_j_*	Freedom *f*	Average deviation *MS_j_*	*F* value	*F* value	Contribution
A (W/C)	4.23 × 10^−3^	2	2.12 × 10^−3^	6.65	*F*_0.01_(2,2) = 99	3.64%
B (Amount of microcapsules)	8.07 × 10^−2^	2	4.04 × 10^−2^	126.92	*F*_0.05_(2,2) = 19	81.16%
C (Preloading rate)	1.31 × 10^−2^	2	6.55 × 10^−3^	20.58	*F*_0.25_(2,2) = 3	12.62%
D (Error)	6.36 × 10^−4^	2	3.18 × 10^−4^	–	–	2.58%
Total	9.87 × 10^−2^	8	–	–	–	–

**Figure 7:**
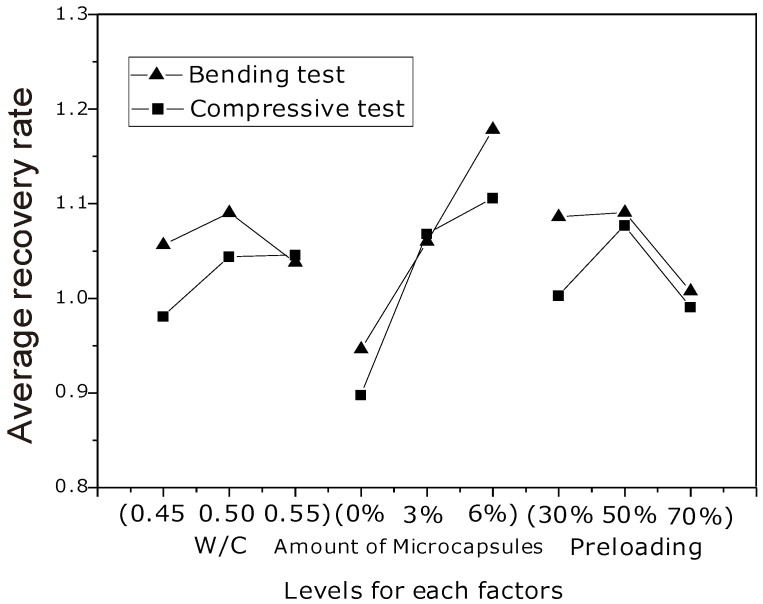
Relationship between the factors and strength recovery rate.

A similar analysis was carried out for the compressive tests and similar results were obtained. Here, only simple curves to guide the eye were given in Figure 7 for both flexural and compressive strength tests. The horizontal axis represented different levels for corresponding factors, while the vertical axis denoted the average recovery rate *K_ij_*. One should see that the average recovery rate was almost proportional to the amount of microcapsules, thus demonstrating that the organic microcapsules could generate self-healing and provide strength recovery, whereas there was no substantial influence from the other factors (preloading and W/C) on the strength recovery rate. It was worthy to mention that, as was seen in Table 2, the strength recovery rates in the specimens without microcapsules of preloading rates being 30%, 50% and 70% were 97%, 101% and 86%, respectively, which seemed a high value. This is especially the case as it is known the original strengths were obtained under continuous loading when it reached peak values. When the load paused at some level for some time, say 30% or 50%, the peak value should not change much from the original one even though there was no autogenous healing process. Whereas higher values of strength recovery rates for the samples with microcapsules should prove the healing effect.

### 2.4. Organic Microcapsules in Mortar Specimens

**Figure 8:**
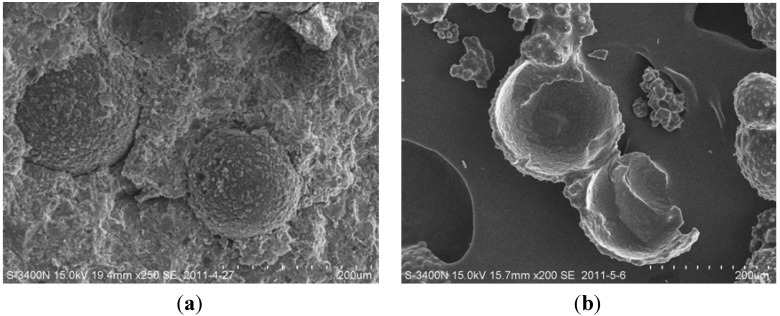
Fracture surface of the specimens. (**a**) Microcapsules combined with mortar matrix and (**b**) Microcapsule ruptured.

After testing, SEM photographs were taken of the fracture surfaces of the specimens, as shown in Figure 8. It was seen that some microcapsules were ruptured under the stress, whereas others were debonded from the mortar matrix.

When a crack approaches a microcapsule, either the microcapsule ruptures, and the healing agent flows out to achieve the healing effect, or debonding occurs (*i.e.*, the microcapsule is debonded from the mortar matrix). In the latter case, no healing occurs, so debonding should be avoided. Hence, under what conditions the two cases occur should be clarified in advance. Theoretical and numerical analysis are prospective to give a criterion for this condition. It has been seen from Figure 7 that the more the amount of microcapsules, the greater the recovery rate was. The strength recovery should come from the adhesion of crack faces which was induced by solidification of flowing out healing agent. Curing reaction mechanism of solidification of (Epoxy/BGE/MC120D) could be referred to [29]. A terpolymer should be obtained.

### 2.5. Chloride Ion Permeability Tests

**Figure 9:**
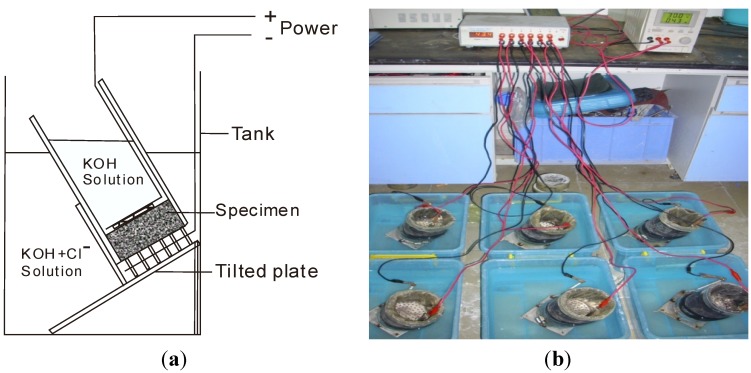
Setup of rapid chloride migration (RCM) test.

**Figure 10:**
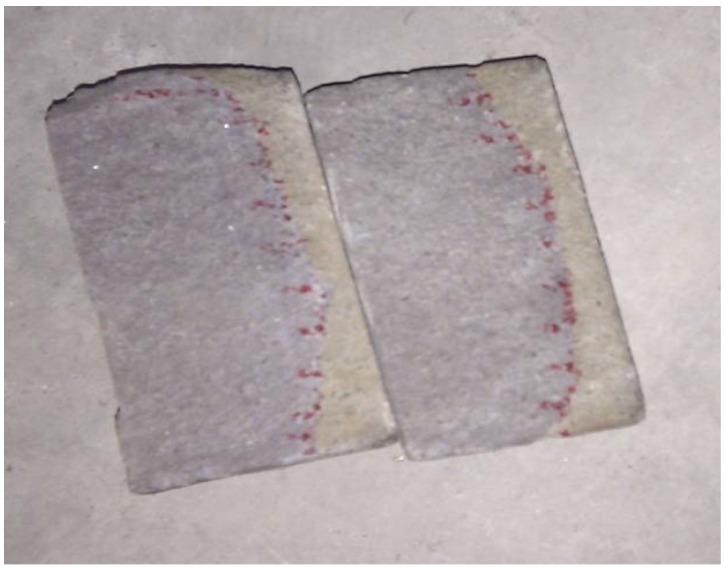
Specimen colored by AgNO_3_ water solution.

To investigate the effect of organic microcapsules on the specimen durability, rapid chloride migration (RCM) tests were carried out [32,33]. The experimental setup is shown in Figure 9. Cylindrical specimens of dimensions 100 mm × 50 mm were used. The water cement ratio and the binder sand ratio were 0.5 and 1:2, respectively. A rubber hollow cylinder surrounded each specimen to isolate the surface of the specimen from water. Inside the hollow cylinder, a 0.2 mol/L KOH water solution (anolyte) was injected. Then, the hollow cylinder was sunk into a mixed water solution (catholyte) (with 5% NaCl and 0.2 mol/L KOH). The base plate was intentionally tilted to release air bubbles generated during electric power switching. For the test, 30.0 V was applied at a temperature of 20–25 °C for 24 h. Then, the specimens were split using a testing machine and colored with a 0.1 mol/L AgNO_3_ water solution, as shown in Figure 10.

Three classes of cylinder specimens, A, B, and C, were prepared with different amounts of microcapsules (0%, 3% and 6% to cement mass, respectively), and each class was divided into four groups,* i.e.*, (1) for strength, (2) for the original chloride ion migration coefficient *D_RCM_*, (3) for *D_RCM_* immediately after preloading, (4) for *D_RCM_* after 3 days of curing (temperature 20 °C, humidity >90%). Three replicates were used in each group. A 50% preloading compression on the specimen was used to generate damage. The load was applied uniformly on the cylinder specimens.

**Figure 11:**
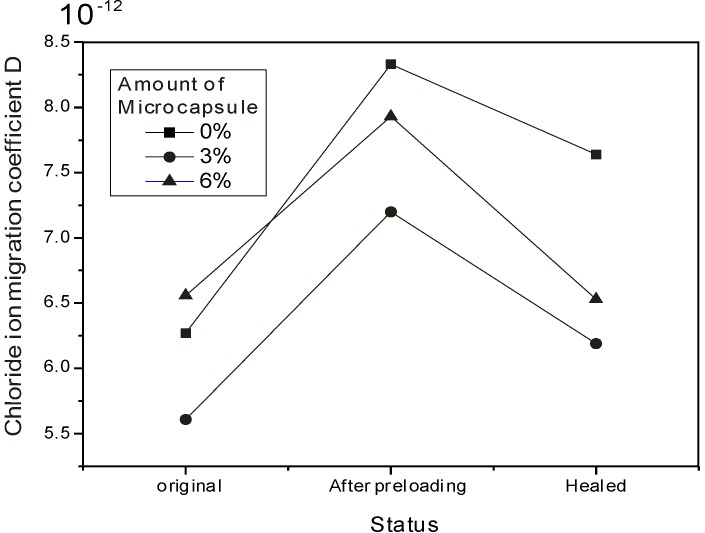
Variation of chloride ion migration coefficient.

The chloride ion migration coefficient was calculated by using [32]:

(5) $$\begin{array}{c} D_{RCM}=2.872\times 10^{-6}\frac{T\cdot h(x_{d}-\alpha \sqrt{x_{d}})}{t}\\ \alpha =3.338\times 10^{-3}\sqrt{T\cdot h} \end{array}$$

where *D_RCM_* is the chloride ion migration coefficient (m^2^/s), *T* is the average temperature of the anolyte (K), *x_d_* is the depth of chloride ion migration (m), *t* is the time during which electricity was applied (s), is a supplementary variable, and *h* is the height of the specimen (m). The test results were shown in Table 4. The calculated chloride ion migration coefficients were shown in Figure 11. One could see that the *D_RCM_* values increased after preloading was applied for all cases, and then decreased after 3 days of curing. An increase of about 20%–30% in *D_RCM_* values after preloading was observed. This should come from the damage and distribution of microcracks generated by the compressive loading. Interestingly, similar to the mechanical behavior, the addition of 3% organic microcapsules leaded to the lowest *D_RCM_* values in the three cases, confirming the microfiller effect from another point of view. A small amount of microcapsules could improve fluidity and compatibility and also generate interfacial defects between microcapsules and the concrete matrix. Certainly, when more organic microcapsules were added, permeability resistance was reduced.

**Table 4:** RCM test results.

No.	Amount of microcapsules	Status	Height of specimen, *h* (mm)	Average temperature, *T* (K)	Depth of chloride ion migration, *x_d_* (mm)
A1	0%	Original	51.3	293.6	14.1
A2	0%	After preloading	55.0	294.1	17.2
A3	0%	Healed	53.1	292.1	16.5
B1	3%	Original	51.4	293.4	12.7
B2	3%	After preloading	51.2	293.4	16.1
B3	3%	Healed	52.8	292.2	13.6
C1	6%	Original	51.2	293.5	14.7
C2	6%	After preloading	51.3	293.7	17.6
C3	6%	Healed	50.4	292.3	14.9

Curing for 3 days improved the permeability resistance for all specimens. It is well known that continuous hydration or recrystallization of cement may generate self-healing in concrete. Further, to investigate the healing effect more clearly, the recovery rate and healing effect were defined as follows based on the coefficient *D_RCM_*:

Recovery rate = *D_original_*/*D_healed_* × 100% (6)

Healing effect = (*D_after preloading_*/*D_healed_* – 1)× 100% (7)

**Figure 12:**
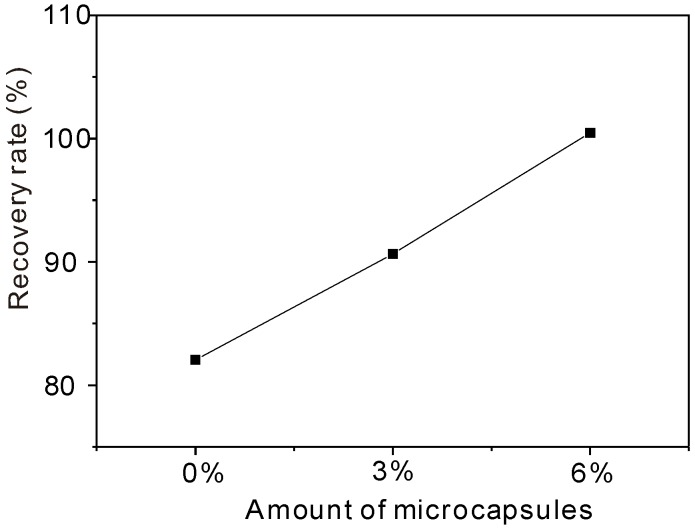
Permeability recovery rate* versus* amount of microcapsules.

**Figure 13:**
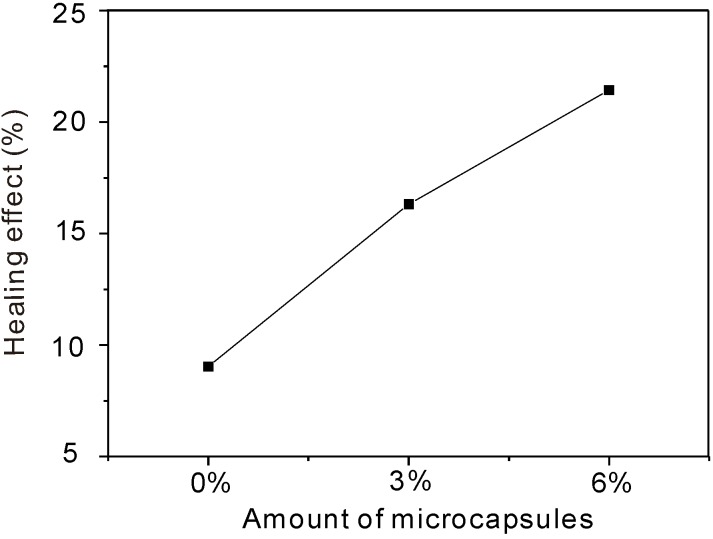
Permeability healing effect* versus* amount of microcapsules.

To understand the definitions easily, two examples can be used to interpret: a 100% recovery rate represents the *D_RCM_* value for the healed specimen’s complete recovery to its original level, whereas 0% healing effect means the *D_RCM_* value for the healed specimen was the same as that immediately after preloading. Figure 12 and Figure 13 show the variation in the recovery rate and the healing effect, respectively, with the amount of organic microcapsules. One can see from Figure 12 and Figure 13 that both the recovery rate and the healing effect are almost proportional to the amount of organic microcapsules. Though mortar itself without microcapsules has a self-healing ability to some extent,* i.e.*, less than a 10% healing effect, the addition of 6% microcapsules could almost recover its original impermeability, and the healing effect increased to over 20%, thus demonstrating the self-healing effect obtained by using organic microcapsules.

## 3. Conclusions

(1) In this study, a novel self-healing cementitious composite was developed using organic microcapsules.

(2) The self-healing performance of the composite was analyzed through its mechanical properties and permeability measurements. The results show that macro properties could indeed be recovered in a self-healing sense.

(3) An orthogonal test was designed and implemented to investigate the influence of three factors (W/C, amount of microcapsules, preloading rate) on the strength recovery rate. It was found that the amount of microcapsules could mostly affect the recovery rate, with the influence of preloading and W/C being relatively weak.

(4) The addition of an appropriate amount of microcapsules in cement materials could reduce the pore content and surface area, which would improve durability and impermeability through a micro-filler effect.
